# The isolation, bioactivity, and synthesis of natural products from *Litsea verticillate* with anti-HIV activities

**DOI:** 10.3389/fphar.2024.1477878

**Published:** 2024-12-09

**Authors:** Jia-Lei Yan, Huiru Nan, Xiaoyu Fang, Xiong-En Long, Yu Jiang, Junyang Liu

**Affiliations:** School of Pharmacy and Food Engineering, Wuyi University, Jiangmen, China

**Keywords:** *litsea verticillate*, natural product, anti-HIV, total synthesis, bioactive

## Abstract

Natural products isolated from *Litsea verticillata* have attracted considerable attention from the chemical community due to their unique structures and promising anti-HIV activities. Recent progresses in the isolation and bioactivity studies for these natural molecules were summarized comprehensively. From the 23 previously uncharacterized compounds isolated from the plant *Litsea verticillata*, litseaverticillol B demonstrated the most potent anti-HIV activity *in vitro*, with IC_50_ ranging from 2 to 3 μg/mL. Meanwhile, litseaverticillol E displayed the highest selectivity index (SI = 3.1), indicating a favorable balance between antiviral potency and cellular toxicity. The plausible biosynthetic pathways and the total synthetic approaches for the representative members (litseaverticillols) were introduced in detail.

## 1 Introduction

Acquired Immunodeficiency Syndrome (AIDS) is caused by infection with the Human Immunodeficiency Virus (HIV) (Fauci, 1988). HIV infection progressively destroys the host’s immune system, leading to severe cellular immunodeficiency and ultimately resulting in serious complications such as wasting, neurological impairment, opportunistic infections, and malignancies (Richman, 2001). Moreover, the emergence of drug resistance mutations (DRMs) in HIV, typically induced by antiretroviral therapy (ART) pressure, can result in rapid virologic failure and limited treatment options (Larder and Kemp, 1989; Tantillo et al., 1994; Lepri et al., 2000), posing a significant public health threat. As a result, the discovery of novel drug candidates or the structural modification of existing drugs has become a critical and urgent priority. In this pursuit, the exploration of natural products has emerged as a prevalent and effective strategy employed by pharmacologists (Li et al., 2024).

Plant-derived natural products are extensively utilized in pharmaceuticals and nutraceuticals, serving as the basis for numerous drugs such as antimalarial artemisinin, analgesic morphine, anticancer paclitaxel, and tanshinones for cardiovascular and cerebrovascular diseases (Atanasov et al., 2015; Newman and Cragg, 2020; Chaachouay and Zidane, 2024). Owing to their broad pharmacological activities, potent efficacy, and significant clinical demand, these natural products have consistently attracted the attention of pharmaceutical chemists (Atanasov et al., 2021; Chopra and Dhingra, 2021; Hu et al., 2024). The *Litsea verticillata* Hance belongs to the genus *Litsea* in the Lauraceae family, which comprises over 400 species that are distributed across southwestern China, Vietnam, Cambodia, Laos, and Thailand (Mabberley, 1997). Notably, *Litsea* species are used in traditional Chinese medicine in China for a variety of therapeutic purposes, such as dispelling wind, removing dampness, and promoting blood circulation (Kong et al., 2015), thereby showcasing considerable medicinal value. The pharmacological activities of *Litsea* species usually encompass antioxidant, antibacterial, anti-inflammatory, cytotoxic, neuroprotective, hepatoprotective, cytokine-modulatory, and analgesic effects (Wang et al., 2016). However, a series of novel compounds with anti-HIV activity were isolated from *L. verticillate* recently, and further investigations into their bioactive constituents are expected to lead to new anti-HIV drugs.

In this review, we focus on the anti-HIV active natural products isolated from *L. verticillata* Hance, discussing their isolation and bioactivities, along with hypotheses concerning their biosynthetic pathways and chemical total synthesis. This review aims to spark the interest of scientists in exploring the bioactive constituents of *L. verticillata*, as well as to encourage pharmacologists to investigate the structural modification of these anti-HIV active compounds that hold promise for clinical drug development.

## 2 Isolation and anti-HIV activities

In 1992, Fong initiated an International Cooperative Biodiversity Group (ICBG) project to investigate *L. verticillata* Hance*,* a perennial shrub or arbor found in the Cuc Phuong National Park, Vietnam (Soejarto et al., 1999). Initial bioassay-directed evaluation of the chloroform extract led to its fractionation through successive flash column chromatography on silica gel and RP-18, followed by preparative high-performance liquid chromatography (HPLC), which resulted in the isolation of a diverse array of active natural products with potent anti-HIV properties (Table 1).

**TABLE 1:** Natural products isolated from *L. verticillata* Hance with anti-HIV activities.

Isolated compound	Separation method	Active part	Anti-HIV to HOG.R5 IC_50_ (μg/mL)	Cytotoxicity to HOG.R5 CC_50_ (μg/mL)	Selectivity index (CC_50_/IC_50_)	References
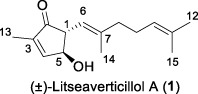	PH	LF + TW	5.0^a^	13.2^a^	2.6	Zhang et al. (2001)
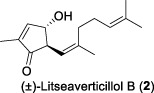	PH	LF + TW	2–3^a^	5.7^a^	2.8–1.9	Zhang et al. (2003a)
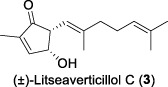	PH	LF + TW	7.1^a^	17.5^a^	2.4	Zhang et al. (2003a)
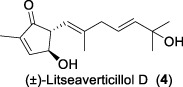	PH	LF + TW	14.4^a^	>20^a^	>1	Zhang et al. (2003a)
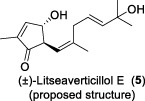	PH	LF + TW	4.0^a^	12.4^a^	3.1	Zhang et al. (2003a)
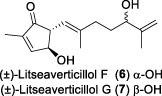	PH	LF + TW	11.3^a^	20.0^a^	1.7	Zhang et al. (2003a)
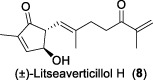	PH	LF + TW	T^a^	2.5–5.0^a^	—	Zhang et al. (2003a)
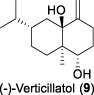	SC	LF + TW	34.5^a^	NT^a^	—	Hoang et al. (2002)
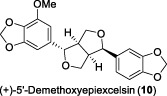	SC	LF + TW	16.4^a^	23.0^a^	1.4	Hoang et al. (2002)
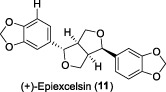	SC	LF + TW	I^a^	NT^a^	—	Hoang et al. (2002)
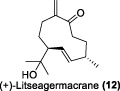	PH	LF + TW	6.5^a^	15.9^a^	2.4	Zhang et al. (2003b)
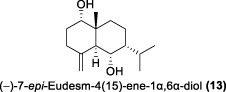	PH	LF + TW	I^a^	NT^a^	—	Zhang et al. (2003b)
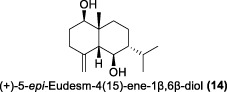	PH	LF + TW	17.4^a^	NT^a^	—	Zhang et al. (2003b)
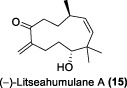	PH	LF + TW	I^a^	NT^a^	—	Zhang et al. (2003b)
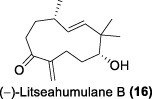	PH	LF + TW	I^a^	NT^a^	—	Zhang et al. (2003b)
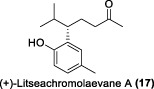	PH	LF + TW	I^a^	NT^a^	—	Zhang et al. (2003b)
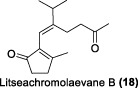	PH	LF + TW	28.0^a^	NT^a^	—	Zhang et al. (2003b)
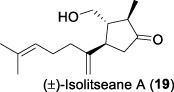	PH	LF + TW	I^b^	NT^b^	—	Zhang et al. (2005)
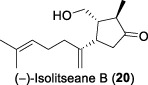	PH	LF + TW	9.0^b^	26.5^b^	2.9	Zhang et al. (2005)
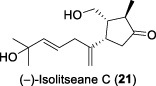	PH	LF + TW	I^b^	NT^b^	—	Zhang et al. (2005)
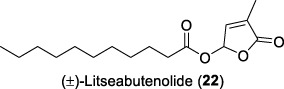	PH	LF + TW	14.0^b^	NT^b^	>1	Zhang et al. (2005)
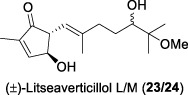	PH	LF + TW + FB	14.0^b^	NT^b^	—	Guan et al. (2016)
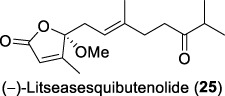	PH	LF + TW + FB	I^b^	-^b^	—	Guan et al. (2016)

*Note*: PH, preparative HPLC; SC, si gel column; LF, leaves; TW, twigs; FB, flower buds; I, inactive; NT, nontoxic at 20 μg/mL; T, toxic.

^a^ Untreated cells served as controls.

^b^ 3TC (Lamivudine) was used as control compound with a IC_50_ value of 1.2 μM in the same HOG.R5 system.

Since 2001, Fong and co-workers isolated a series of active sesquiterpenes with a novel and unique skeleton from the leaves and twigs of *L*. *verticillata* and named these compounds litseanes (Zhang et al., 2001). The first sesquiterpene identified with anti-HIV-1 replication properties was named as litseaverticillol A (**1**). It was a racemic sesquiterpenoid with a unique structure that had not been reported previously. The inhibitory activity against HIV-1 was conducted using HOG.R5, a reporter cell line developed to quantify the replication of HIV-1. The results indicated that litseaverticillol A suppresses HIV-1 replication with an IC_50_ value (the concentration that inhibits viral replication by 50%) of 5.0 μg/mL (21.4 μM). However, this compound also showed cytotoxic effects on HOG.R5 cells, with a CC_50_ value (the concentration that reduces cell viability by 50%) of 13.2 μg/mL (56.4 μM).

In 2003, Fong and co-workers reported the isolation and structural elucidation of eight previously undescribed litseane sesquiterpenes, designated as litseaverticillols B-H (**2**-**8**), from the leaves and twigs of *L. verticillate* (Zhang et al., 2003a). The stereochemical configurations of these compounds were determined to be racemic mixtures, as confirmed by Mosher ester reactions and optical rotation analyses. The structures of litseaverticillols B–H were determined to be closely related to that of litseaverticillol A. These compounds exhibited IC_50_ values ranging from 2 to 15 μg/mL (8–58 μM) in the HOG.R5 cell-based system. Primary studies on the structure-activity relationship of these compounds indicated that the *Z*,*E* geometrical configuration at ∆^6,7^, the chirality at the C1 position, and the position of the double bonds at C9–C10, C10–C11, and C11–C12, all influence the anti-HIV activity of the compounds. For instance, compared to **6**/**7**, compound **8** exhibited significantly enhanced anti-HIV activity, attributed to the presence of an α,β-conjugated structure at the C-10 position and the ∆^11,12^ ring system. However, compound **8** displayed noticeable cytotoxicity, making it unsuitable for evaluation as an anti-HIV drug. In Fong’s study, litseaverticillols B-H (**2**-**8**) exhibited CC_50_ values in HOG.R5 cells, that were 2–3 times higher than their corresponding IC_50_ values, resulting in a selectivity index (SI = CC_50_/IC_50_) ranging from 2 to 3 for these compounds. While this selectivity index is not optimal for an ideal drug candidate, the valuable structure-activity relationships observed for the litseaverticillol series provide valuable insights for the exploration of new drug candidates.

Lignans possess a unique chemical architecture, often consisting of two phenylpropane units linked by a carbon-carbon bond. This core framework frequently adopts diverse configurations, such as dibenzylbutanes, dibenzylbutyrolactones, and aryltetralin lignans. Additionally, lignans often contain multiple hydroxyl groups attached to the aromatic rings, which enhance their antioxidant potential. These structural elements endow lignans with a wide array of biological activities and considerable pharmacological promise (MacRae and Towers, 1984; Xiao et al., 2023; Liang et al., 2024).

In 2002, Fong and co-workers discovered a sesquiterpene and two lignans in *L. verticillata* Hance (Hoang et al., 2002). These compounds were identified as verticillatol (**9**), (+)-50-demethoxyepiexcelsin (**10**), and the known compound (+)-epiexcelsin (**11**), respectively. (+)-epiexcelsin (**11**) differs from verticillatol (**9**) only in the presence of a methoxy group at position 5'. Compounds **9**-**11** were evaluated for their *in vitro* inhibitory effects against HIV replication in HOG.R5 cells. Compound **10** exhibited strongest selective anti-HIV-1 activity, displaying an IC_50_ value of 16.4 μg/mL (42.7 μM). However, its selectivity index (SI = 1.4) was deemed unsuitable for further development. Compound **11** exhibited no inhibitory activity against HIV-1 replication, potentially attributed to the presence of the 5′-methoxy group and its reduced solubility relative to Compound **10**. The eudesmane sesquiterpenoid (**9**) exhibited modest activity against HIV-1, with an IC_50_ value of 34.5 μg/mL (144.7 μM). Notably, this compound showed no toxicity up to a concentration of 20 μg/mL. This represents the inaugural identification of sesquiterpenoids within this class demonstrating anti-HIV activity.

The mechanism underlying lignans’s anti-HIV activity entails inhibiting viral replication by interfering with multiple stages of the viral life cycle, encompassing viral entry, reverse transcription, integration, and assembly (Rimando et al., 1994). Moreover, lignans can modulate host immune responses, inhibit key viral enzymes like reverse transcriptase and protease, or disrupt critical viral protein-protein interactions necessary for replication (Hara et al., 1997). These multifaceted mechanisms collectively enhance the antiviral efficacy of lignans against HIV.

In the same year, Fong and co-workers isolated seven new sesquiterpenes from *L. verticillata*, designated as litseagermacrane (**12**), 7-*epi*-eudesm-4 (15)-ene-1α,6α-diol (**13**), 5-*epi*-eudesm-4 (15)-ene-1β,6β-diol (**14**), litseahumulanes A (**15**) and B (**16**), and litseachromolaevanes A (**17**) and B (**18**) (Zhang et al., 2003b). Three compounds (**12**, **14**, **18**) demonstrated moderate to weak anti-HIV activity. litseagermacrane (**12**) exhibited the highest anti-HIV activity, with an IC_50_ value of 6.5 μg/mL (27.5 µM), but it also displayed cytotoxicity to HOG.R5 cells with a CC_50_ value of 15.9 μg/mL (63.4 µM). The IC_50_ values of isolates **14** and **18** were 17.4 μg/mL (73.1 µM) and 28.0 μg/mL (119.7 µM), respectively, with no cytotoxicity observed up to a concentration of 20 μg/mL. However, the selectivity index (SI) values for isolates **12**, **14**, and **18** indicated low selectivity.

In 2005, the research team led by Tan and Zhang continued their phytochemical investigations on the leaves and twigs of *L. verticillata* Hance, leading to the isolation of eleven compounds (Zhang et al., 2005). The isolated compounds included three previously undescribed sesquiterpenes, isolitseanes A-C (**19-21**), in addition to a new butenolide derivative, litseabutenolide (**22**). The isolitseane sesquiterpenes A, B, and C exhibited subtle structural differences. Isolitseanes A and B differed only in the configuration of the C6 stereogenic center. In contrast, isolitseane C possessed the same pentacyclic ring system as isolitseane B, with minor structural variation at the position of double bond and oxidation state of the side chain. Litseabutenolide (**22**) was characterized as a furan-containing ester. Subsequent HIV-1 antiviral evaluation studies demonstrated that isolates **20** and **22** inhibited HIV-1 replication in HOG.R5, with IC_50_ values of 38.1 and 40.3 μM, respectively.

In 2016, Zhang and co-workers discovered three additional novel sesquiterpenes from *L. verticillata*, namely, litseaverticillols L and M (**23/24**) and litseasesquibutenolide (**25**) (Guan et al., 2016). Litseaverticillols L and M (**23**/**24**) exhibited anti-HIV activity with an IC_50_ value of 49.6 μM and no toxicity to host HOG.R5 cells at a concentration of 70 μM. These findings indicated that litseaverticillols L and M (**23**/**24**) possess potency comparable to that of litseaverticillol D (**25**) and other litseanes. However, litseasesquibutenolide (**25**) did not show inhibitory activity against HIV-1 replication at a concentration of 70 μM.

In summary, most natural products isolated from *Litsea verticillata* exhibit good anti-HIV effects, whereas litseaverticillols display exceptional inhibitory activity in both anti-HIV efficacy and selectivity indices. Of note, lignans are the most abundant compounds produced in this plant, and litseanes are considered minor components, with litseaverticillol A being the most abundant at a yield of 0.0016% (Guan et al., 2016). Among them, litseaverticillols B (**2**) and E (**5**) exhibited the best anti-HIV efficacy with IC_50_ = 2-3 and 4.0 μg/mL, respectively, but their selectivity indices (CC_50_/IC_50_) remain inadequate for therapeutic applications. Moreover, both litseaverticillols B (**2**) and E (**5**) were isolated as racemic mixtures, and the activities of their optically pure forms have yet to be determined. Consequently, further investigations on the total synthesis, structure modification, and activity evaluation of these natural products are highly demanded.

## 3 Biosynthetic pathways

**FIGURE 1:**
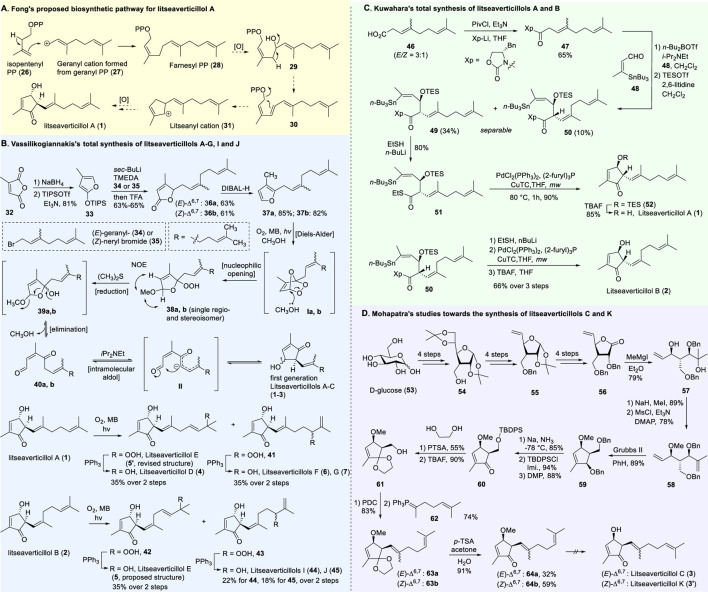
**(A)** The biosynthetic pathway for litseaverticillol A proposed by Fong; **(B)** The total synthesis of litseaverticillols A–G, I and J by Vassilikogiannakis’s group; **(C)** The total snythesis of litseaverticillols A and B by Kuwahara’s group; **(D)** Synthetic studies towards litseaverticillols C and K by Mohapatra’s group.

A plausible biosynthetic pathway for litseaverticillol A (**1**) was proposed by (Zhang et al., 2001). As displayed in Figure 1A, litseaverticillol A was probably formed through the mevalonate pathway characteristic of sesquiterpenes. Given that the side chain of litseaverticillol A (**1**) represents a geranyl unit, it was postulated that the condensation of an isopentenyl diphosphate (**26**) with a geranyl diphosphate derived cation **27** produced the farnesyl diphosphate (**28**). Intermediate **28** was then oxidized to **29**, followed by a sequential cyclization and oxidation to afford the litseaverticillol A (**1**). As far as we know, this remains the only reported example of biosynthetic investigation for natural products belonging to this family.

## 4 Total synthesis

Due to their notable bioactivity and unique chemical structure, natural products from *Litsea verticillate* have become attractive targets in the field of synthetic chemistry (Vassilikogiannakis and Stratakis, 2003). Thoroughly analyzed the structure of litseaverticillols, and concluded that litseaverticillols D-H (**4**-**8**) could arise from direct oxidation of the litseaverticillols A-C (**1**-**3**) via an ene reaction with ^1^O_2_ at the side-chain double bond most distal to the cyclopentenone ring. Thus, a divergent synthetic strategy was employed in their synthetic work (Vassilikogiannakis and Stratakis, 2003; Vassilikogiannakis et al., 2004; Vassilikogiannakis et al., 2005; Margaros et al., 2006). As shown in Figure 1B, the synthesis started from commercially available citraconic anhydride (**32**), which was then transformed to 2-triisopropylsilyloxyfuran (**33**) in two steps. *Ortho*-lithiation at the 5-position of **33** with sec-butyllithium, followed alkylatioin of the resultant anion with either geranyl (**34**) or neryl bromide (**35**) and subsequent acidic hydrolysis of the triisopropylsilyl protection group, afforded lactones **36a** and **36b** in moderate yields, respectively. DIBAL reduction of lactones **36a** and **36b** furnished sesquirosefuran **37a** and **37b**, respectively. Subsequently, a key singlet oxygen (^1^O_2_) cascade sequence was employed to derive the complete litseaverticillol core. This cascade sequence was required to initiate at the furan moiety without concomitant side reaction at either of the two susceptible double bonds in the attached side chain. Under the optimized conditions, sesquirosefuran **37a/b** was irradiated for 1 min in a methanolic solution (containing 10^−4^ M methylene blue as a photosensitiser) with O_2_ gently bubbled through it, resulting in the exclusive formation of hydroperoxides **38a**/**b** as the [4 + 2] adducts in quantitative yields. Afterward, hydroperoxide **38a/b** was reduced by dimethyl sulfide, followed by an elimination of methol and subsequential intramolecular aldol reaction, to produce the first generation litseaverticillols A-C (**1**-**3**). Then ^1^O_2_-mediated oxidation of the distal double bond (△^10,11^) in the side chains of litseaverticillol A (**1**) provided litseaverticillol E (**5′**, revised structure) and peroxide **41**, which were reduced by triphenylphosphine to litseaverticillol D (**4**) and litseaverticillols F (**6**), G (**7**), respectively. Similarly, litseaverticillol B (**2**) can be transformed to litseaverticillol E (**5**, proposed structure) and litseaverticillols I (**44**), J (**45**), respectively. Notably, litseaverticillols F (**6**)/G (**7**) and litseaverticillols I (**44**)/J (**45**) were all separated by flash chromatography.

The first enantioselective total synthesis of litseaverticillols A and B was disclosed by (Morita et al., 2006; Morita and Kuwahara, 2006). Therein an Evans asymmetric aldol reaction and a microwave-promoted cyclization of a stannylated thiol ester intermediate were employed as the key C−C bond-forming steps. As displayed in Figure 1C, the readily available acid **46** (a 3:1 *E*/*Z* mixture) was first converted to the oxazolidinone derivative **47**, which then reacted with aldehyde **48** via the *syn*-selective Evans aldol reaction to produce two separable aldol products **49** and **50** in 34% and 10% yields, respectively. The *E*-isomer **49** was transformed to the thiol ester **51**. Subsequently, the microwave-promoted cyclization of **51** was achieved in the presence of PdCl_2_(PPh_3_)_2_, (2-furyl)_3_P and CuTC, leading to compound **52** in 63% yield. After the desilylation of **52**, (1*R*,5*S*)-litseaverticillol A was afforded in 85% yield. By following the similar reaction sequences, the *Z*-isomer of aldol product **50** was converted to (1*R*,5*S*)-litseaverticillol B (**2**) in 66% yield over the three steps.

The first enantioselective synthesis towards the litseaverticillols C and K was reported by (Mohapatra et al., 2007). The synthesis utilized the ring-closing metathesis (RCM) and Wittig reactions as key steps. As shown in Figure 1D, the starting material, D-glucose (**53**), was converted to lactone **56** in 12 steps involving oxidation state adjustment and functional group transformations. Lactone **56** was then subjected to Grignard addition to produce alcohol **57**, which then underwent an *O*-methylation and a base-promoted Hoffmann elimination to give diene derivative **58**. Ring-closing metathesis of **58** proceeded effectively to give the cyclopentene core **59**, which was subsequently converted to enone **60** through a three-step sequence involving (1) reductive cleavage of the benzyl ethers, (2) selective protection of the primary hydroxyl group, and (3) oxidation of the allylic alcohol. After a two-step protection group transformation, the resultant **61** was oxidized to aldehyde, and then subjected to Wittig reaction with ylide **62** to give a mixture of *E*- and *Z*-isomers (**63a**/**63b**) in 35:65. Subsequent deketalisation of the **63a**/**63b** mixture under acidic conditions furnished keto derivatives **64a**/**64b**, which were separated by silica gel chromatography. Finally, hydrolysis of the methyl ether at C-1 of **64a**/**64b** would produce the desired litseaverticillols C and K. Unfortunately, the hydrolysis reaction was unsuccessful under various conditions.

## 5 Summary and outlook

Due to their unique structures and promising anti-HIV activities, natural products derived from *L. verticillata* have garnered significant interest from the chemical community. Among these, litseaverticillols serve as representative molecules and have been extensively studied for their bioactivity. Several natural molecules of this family exhibited potent anti-HIV activities, but also inhibited the growth of human cells (HOG.R5), leading to the selectivity indices (SI = CC_50_/IC_50_) for the most promising compound in the range of 2–3. This underscores the need for structural optimization to identify new drug leads. Inspired by the plausible biosynthetic pathways, three research groups have developed diverse synthetic approaches to access the litseaverticillols. Despite these pioneering efforts, there is still a need for more efficient synthetic approaches with high stereoselectivities. Furthermore, analog production and structure-activity relationships (SAR) analysis are required as well to explore new anti-HIV lead compounds. Looking ahead, we anticipate expansive research into the isolation, synthesis, and bioactivity studies of these natural products. It is hoped that this review will provide useful information for future research efforts on natural products from *Litsea verticillate*.
